# Disentanglement of Surface and Confinement Effects for Diene Metathesis in Mesoporous Confinement

**DOI:** 10.1021/acsomega.3c06195

**Published:** 2023-12-19

**Authors:** Ingo Tischler, Alexander Schlaich, Christian Holm

**Affiliations:** † Institute for Computational Physics, University of Stuttgart, 70569 Stuttgart, Germany; ‡ Stuttgart Center for Simulation Science, University of Stuttgart, 70569 Stuttgart, Germany

## Abstract

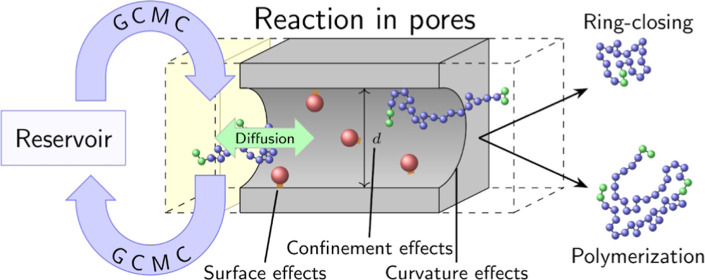

We study the effects of a planar interface and confinement on a generic catalytically activated ring-closing polymerization reaction near an unstructured catalyst. For this, we employ a coarse-grained polymer model using grand-canonical molecular dynamics simulations with a Monte Carlo reaction scheme. Inspired by recent experiments in the group of M. Buchmeiser that demonstrated an increase in ring-closing selectivity under confinement, we show that both the interface effects, i.e., placing the catalyst near a planar wall, and the confinement effects, i.e., locating the catalyst within a pore, lead to an increase of selectivity. We furthermore demonstrate that curvature effects for cylindrical mesopores (2 nm < *d* < 12.3 nm) influence the distribution of the chain ends, leading to a further increase in selectivity. This leads us to speculate that specially corrugated surfaces might also help to enhance catalytically activated polymerization processes.

## 1 Introduction

As 80% of the global industry relies on catalysis,^1^ catalytic processes can be considered an essential part of the global industry. Therefore, there is a demand for developing new or improving existing catalytic methods. One way to achieve the latter is to mimic the behavior of biocatalysts. These systems rely on enzymes, which are usually faster and more stable than molecular catalysts.^2^ Furthermore, these biocatalysts were tailored by evolution to react in a specific pathway, which makes them highly selective toward the desired reaction products. One reason for the preferred catalytic behavior is that the enzyme encloses the substrate and forces the reaction to take place in a localized confined space. Inspired by such confinement-induced effects, chemical engineers have utilized zeolites^3,4^ or metal–organic frameworks^5,6^ for heterogeneous catalysis and cyclodextrins^7^ or self-assembled container molecules^8,9^ for homogeneous catalysis.

Another approach to utilizing confinement effects is by anchoring a molecular catalyst inside a mesopore. An example reaction used in industry^10,11^ and for pharmaceutical applications^12^ is the olefin metathesis,^13^ which swaps the partners within a pair of carbon–carbon double bonds. Applying this catalysis to diene oligomers results in two reaction pathways: a ring-closing reaction and a polymerization. The difference between them is that for the ring-closing reaction, the pair of double bonds are from the same molecule, while for the polymerization, they come from two different ones. Because both reactions are possible, we have a ring–chain-equilibrium,^14^ where the selectivity toward the ring-closing can be defined as
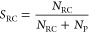
1with *N* being the number of ring-closed (RC) or polymerized (P) molecules produced. This ring–chain equilibrium depends on the enthalpy and entropy of the system. While for shorter chains, the ring strain is the dominating energy contribution, for longer chains, this can be neglected due to their higher flexibility.^15^ To control the selectivity of the macrocyclization process,^16^ concentration variations can be used. Upon concentration increase, more olefins can collide with the catalyst, hence favoring polymerization. Vice versa, lowering the concentration enhances the ring-closing selectivity at the price of slowing the reaction. For upscaling to industrial and pharmaceutical processes,^17,18^ the ring-closing selectivity needs to be increased while running the catalysis at high substrate concentrations. One way to overcome this limitation is to develop more stable catalysts that live long enough to disassemble the polymerized products into RC products via so-called backbiting.^19,20^

Another approach employs confinement to restrict the movement of the dienes, which should make it easier to form rings.^21,22^ Ziegler et al.^23,24^ showed that in this way, the ring-closing selectivity increases significantly. For certain substrates, they observed a rise from *S*_RC_ = 19% to *S*_RC_ = 59% when the catalyst was immobilized in silica mesopores with a diameter between 2.5 and 6.1 nm. While these experiments showed the benefits of employing the confinement effects, the underlying mechanisms and their interplay remain to be understood in detail.

In an earlier study,^25^ we could show via random walk theory and molecular dynamics (MD) simulations that the presence of a flat wall near the catalyst has positive feedback on the ring-closing probability. Here, we study the confined ring-closing metathesis reaction on a coarse-grained level. On this scale, we can analyze diffusion processes into and out of the pore for the different reaction species. We are also able to investigate and separate the different effects at play. To this end, we developed a coarse-grained reaction model of the metathesis reaction. The model operates similarly to the interacting-particle reaction dynamics approach by the software package ReaDDy,^26,27^ where triggered discrete reaction events cause either the creation or destruction of bonds. It also compares to the reactive MD model by Akkermans et al.,^28^ which is used to study polymerization processes.^29−31^

We have simulated mesoscopic slits and cylindrical pores with diameters of 2.0 nm < *d* < 12.1 nm. Since we want to distinguish between surface effects and confinement effects, we investigated systems where the catalyst is fixed in pores of different sizes, or alternatively, at flat walls where only surface effects are present. Additional effects can appear in cylindrical pores due to curvature. With the help of these simulations, we are therefore able to distinguish between wall, confinement, and curvature effects.

This article is structured as follows. We begin with the description of the methods where the model will be introduced, and the systems that are being simulated are explained. The results of these simulations are presented and discussed in the section that follows. The last section is devoted to the conclusions.

## 2 Methods

We employ a coarse-grained model using the ESPResSo MD software package.^32^ In our simulations, the substrate oligomers are represented by Kremer–Grest polymers,^33,34^ which repel via a WCA potential and attract via a FENE potential
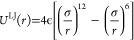
2
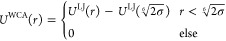
3
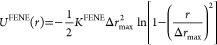
4Here, ϵ and σ define the length and energy scales of the WCA potential, *r*_max_ defines the distance where the bonded potential diverges, and *K* is the strength of the FENE potential. Our Kremer–Grest polymer model is an implicit solvent model that together with the Langevin thermostat yields polymer statistics under good solvent conditions. For simplicity, we considered only linear chains consisting of two different numbers of monomers, *N*_m_ ∈ {22, 29}. The number of monomers of these oligomers was chosen to resemble substrates 1 and 4 in the experiments by Ziegler et al.,^24^ since these resemble reasonably well a linear polymer chain. To include the rigidity of the molecule, we added a harmonic angular potential between the individual beads that has an equilibrium angle of ϕ_0_ = 180°
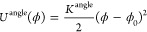
5

The strength of this potential, *K*^angle^ is a free parameter of the model used to fit the selectivity of our homogeneous reaction to the experimental values. For the bond angle potential of our polymer model, we determined *K*_22_^angle^ = 5.09*k*_B_*T* and *K*_29_^angle^ = 4.32*k*_B_*T* to yield sufficient agreement with the experimental data (see Supporting Information, Figure S1). With this stiffness, the hydrodynamic radii of our substrate oligomers were calculated according to Doi and Edwards^35^
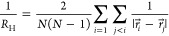
6

For the longer chain, we obtained *R*_H29_ = 0.59 ± 0.01 nm, while the experimental value is *R*_H29_^ex^ = 0.53 nm. For the shorter chain, we compare the simulated value of *R*_H22_ = 0.51 ± 0.01 nm to the experimentally determined value of *R*_H22_^ex^ = 0.44 nm. With respect to the coarse-grained level of our simulations, we found the agreement sufficient.

### 2.1 Metathesis Model

**Figure 1:**
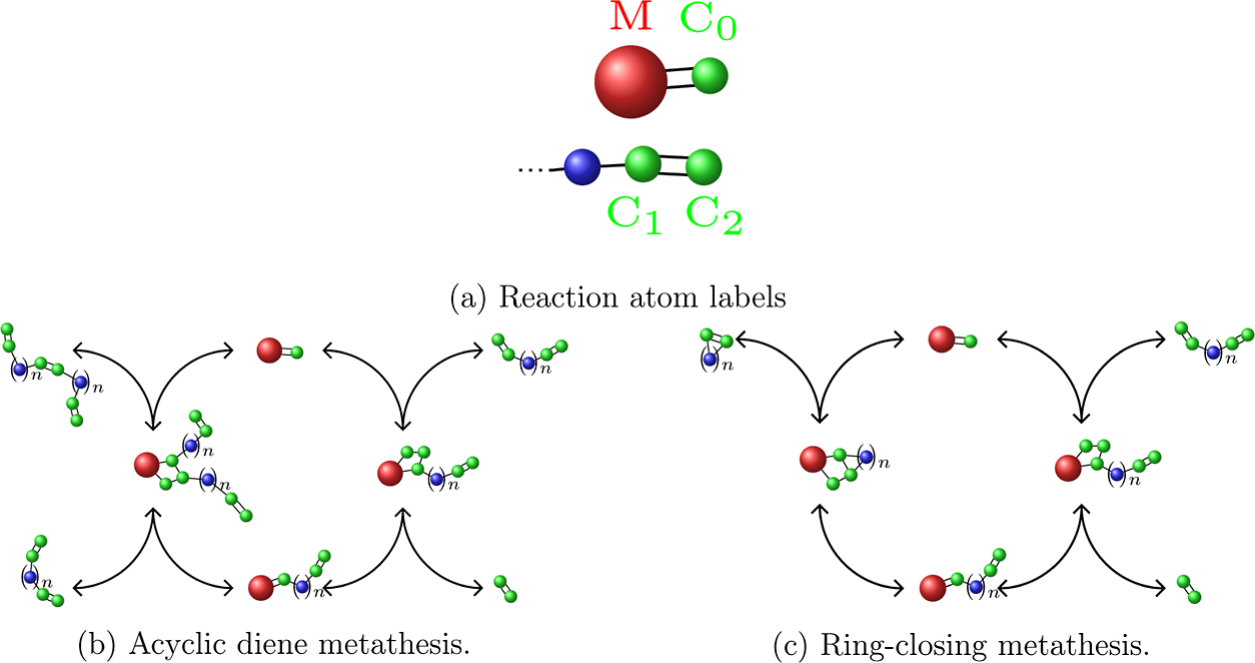
Scheme of different pathways for a diene metathesis reaction. The catalyst is shown in red; the green particles denote those carbon atoms that share a double bond. Only these can attach to the catalyst. The other backbone carbon atoms are depicted in blue. Number of backbone atoms for our case is *n* ∈ {18, 25}. In (a), the labeling of the reaction participant particles is shown. In (b), we see the different pathways of an acyclic diene metathesis. In (c), we observe a ring-closing metathesis. In the center-top and center-bottom stages, the catalyst can accept a bond from the green particles. In the center left of the picture, the bonds have been formed. Reactions can either continue forward (clockwise) or backward (counterclockwise). This depends on the order in which the bonds will break. The right path for both reactions is the same up to the point where either (b) a carbon chain is attached to the catalyst or (c) the two ends of the chain close onto themselves to form a ring.

The reaction mechanism^36,37^ of the olefin metathesis we want to mimic is the following: a metal atom is located in the center of the catalyst complex. This metal atom is the central part of the reaction and can form or break bonds with the substrate molecules. We assume that initially, the metal atom has already formed a double bond with a carbon atom, which we will call C_0_. Substrate carbon atoms that share a double bond can attach to the catalyst and are denoted by C_1_ and C_2_ (see Figure 1a). When C_1_ binds to the catalyst, the double bond between C_1_ and C_2_ is reduced to a single bond. The same happens to the bond between the metal atom and C_0_. To keep the total number of bonds constant, C_0_ and C_2_ also form bonds. Thus, C_0_, C_2_, and C_1_ and the metal atom have formed a 4-fold ring. Two carbon atoms can then split off from this ring so that the carbon pairs C_0_ and C_2_ are freed and C_1_ remains bonded to the metal atom. During this reaction, the bonding partners of the substrate have swapped double bonds. This process does not interfere with any other chains attached to the carbon atoms.

In our coarse-grained approach, we model the catalyst as a large particle that has two active sites on its surface, which can form the two bonds that the metal atom can accept. If one of the active sites already has a bond, then it will be deactivated until it releases the bond particle. The substrate molecules are simple bead–spring chains in which each bead reflects a carbon atom. The first two and last two monomers of each chain are labeled as the carbon atoms that share a double bond. These labeled carbon atoms can form a bond with the catalyst once they collide with the active site. The probability that this bond will be accepted is called *P*_bond_. The carbon atom of a pair that collides first obtains the bond, while the other forms a bond with the particle that was bonded to the catalyst before the reaction. These bonds formed during the reaction process are treated as harmonic bonds because the distance between the particles when the bonds are created can vary strongly, which can lead the FENE bonds to be overextended. The such created 4-fold ring can break open to release another pair of labeled particles. This breakup is triggered at a predefined rate τ_break_^–1^. During the breaking process, if C_0_ and C_2_ remain bonded together, the harmonic bond between them is replaced by a FENE bond to be consistent with the rest of the oligomer.

Although this method technically allows for modeling a generic metathesis reaction, we applied it here to the specific case of an α,ω-diene metathesis reaction. In that case, there are two opposite reaction paths of ring-closing and polymerization. In both cases, a substrate molecule is already bound to the catalyst, and the reaction pathway depends on whether the next metathesis process starts with the olefin bond on the other side of the molecule or from a completely different substrate molecule. A schematic picture of how this diene metathesis proceeds in the model can be seen in Figure 1. Technically, it is also possible for the olefin bond to attach with C_1_ and C_2_ atoms being swapped, but this leads only to an unproductive reaction cycle in which only the carbon atom bonded to the catalyst is exchanged and the reaction continues as if this has not happened.

While this simple model is subject to some limitations, many of them can be overlooked, as we are only interested in modeling the selectivity of the reaction. First, we ignore the inconsistencies in the binding energies of the different molecules during the reaction. When a bond is formed, a pair potential is created between two particles that may not be in equilibrium, causing a change in the local energy. However, due to the thermalization of the entire system, this is quickly dissipated. Additionally, the driving force of the reaction stems more from the entropy of the created ethylene than from the enthalpy change due to the negligible ring strain of macrocycles. Second, since we are not interested in the kinetics of the reaction itself, we accelerate the reaction by increasing the breakage rate of the 4-membered ring and also by increasing the probability of accepting the bond upon collision to *P*_bond_ = 1. This is done such that reaction and diffusion occur on the same time scale. This acceleration of the reaction should affect only the throughput and not the selectivity. Contrary to the experiments, our catalyst is an ideal catalyst that is infinitely stable. However, for our simulations, we use a GCMC reservoir, which controls the concentration of the reactants, to emulate a system state in which the reaction is always in its early stages. Next, the catalyst is modeled as a sphere and neglects the ligands that strongly affect the reaction. While the ligands usually play an important role in defining the catalytic properties, we have neglected these properties in the development of our model. Also, finally, due to our coarse-grained polymer model, we also avoided the topic of stereoselectivity. These limitations have only a minor effect on the selectivity and are overcome by the parametrization of the bond angle potential mentioned earlier.

### 2.2 GCMC Reservoir

From the experiments, there are two important conditions that we would like to keep in mind in our simulational approach: first, the reaction takes place within the confinement of the pore and second, the diffusion processes into and out of the pore are handled accordingly. As the reaction is addressed as described above, we now mimic the diffusion of the substrate and product in and out of the pore. To model the diffusion process, we consider a finite porous media (i.e., the interface, the slit, or the cylindrical pore) embedded in a reservoir where the reactants are dissolved in. This reservoir region is treated via the grand canonical Monte Carlo (GCMC) technique.^38^ Thus, substrates can be introduced into the system and products can be removed from it. GCMC is a simple technique to impose the fixed chemical potential of a reservoir on the system of interest, i.e., the pore. It works by removing or inserting a molecule via a trial move. The probability  for accepting this move is given by the Boltzmann-coefficient, which results in
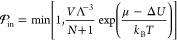
7and
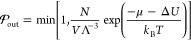
8where *V* is the volume of the system, Λ is a normalization factor, *N* is the number of molecules in the system prior to the move, μ is the desired chemical potential, and Δ*E* is the energy difference of the insertion/removal moves, which are accepted according to a Metropolis scheme. For our simulation, we remove the products from the reservoir region of the system by setting their chemical potential to μ_product_ = −∞. This circumvents the problem that one must specify the composition of the products when handling them with a finite chemical potential. To account for diffusion effects, we define a specific region around the catalyst that is not coupled to the reservoir, which means that the molecules must diffuse toward and away from the catalyst. The latter allows counting the ratio of polymerized/RC molecules to determine *S*_RC_. Following this protocol, we simulate the reaction in a steady state, mimicking the experimental situation at the beginning of the reaction.

### 2.3 System Setup

In order to study the various effects when confining a system, we investigated a total of four different realizations, with varying levels of confinement: bulk, a flat wall, slit pores, and cylindrical pores. The bulk system represents homogeneous catalysis without any confinement. A flat wall with periodic boundary conditions is used to study surface effects with an otherwise open system. The slit pore goes further in the sense that it confines the catalyst on both sides. By varying the slit width, we can therefore control the contribution of this confinement effect. Finally, using a cylindrical pore with a variable radius, we also add the effect of curvature to our study.

**Figure 2:**
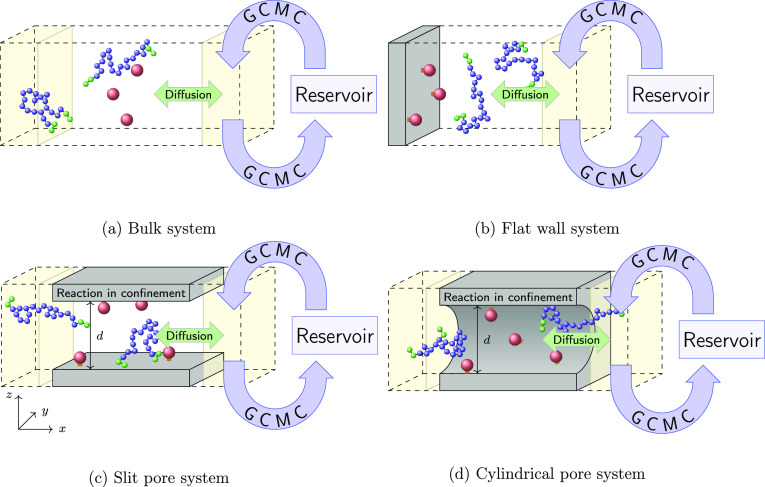
Illustration of the different systems. In all systems, the catalysts are fixed in space such that the reactants must diffuse to them. Only the areas highlighted in yellow are coupled to the reservoir, where the chemical potential is imposed. Substrates are introduced into this coupled volume and products are withdrawn. System itself is periodic in all directions.

The bulk system is depicted in Figure 2a. The positions of the catalysts are fixed within the system box. This was done to suppress possible catalyst–catalyst interactions. The spatial region that couples to the reservoir has some distance from the catalysts. This is similar to homogeneous reaction experiments. In the experiments of Ziegler et al., a catalyst loading of 1 mol % is used, meaning that the catalyst/substrate ratio is 1:100. However, due to the influx of substrate particles from the reservoir, the catalyst loading cannot be rigorously defined for our system. Therefore, we used the catalyst density as a free parameter, and after some testing, it turned out not to have a significant impact on the selectivity as long as the catalysts were far enough apart such that the attached chains did not interact with one another. The resulting catalyst density that we chose was at an average substrate density of ρ = 0.015 nm^–3^.

**Figure 3:**
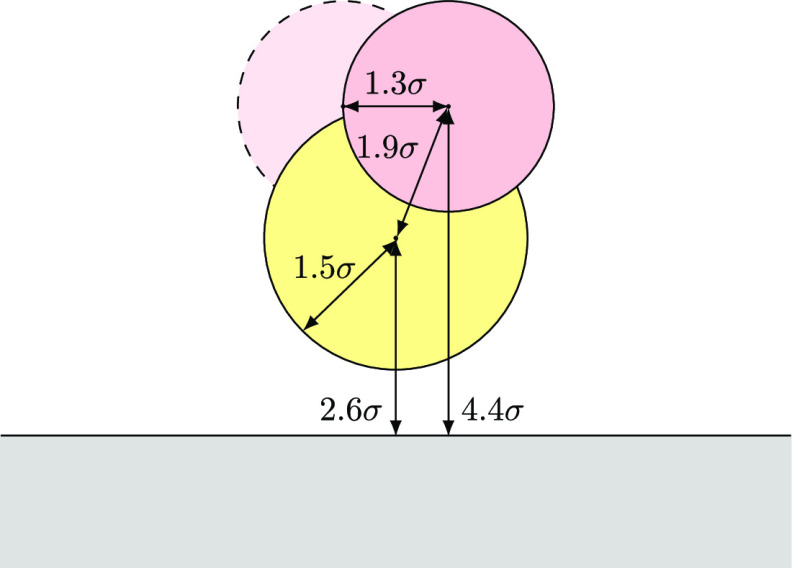
Illustration of the catalyst geometry. All length scales are given with respect to the diameter of our carbon atoms: σ = 1.535 Å. The yellow sphere is the impenetrable holder of the reactive sites (red). The pale, dashed one resembles the inactive reaction side, which already has a particle bond to it. Reactive sites are 1.3σ of each other. These reactive sites are permeable, but if the center of a particle with an olefin bond passes into this region, it will attach to the reactive site. During the reaction, it is impossible for both reactive sites to be active at the same time.

In the other systems, the catalyst is placed near a wall. An illustration of the catalysts near a wall can be seen in Figure 3. The catalyst consists of a central impenetrable sphere, to which two permeable active sites are attached. The position and rotation of the catalysts are fixed. The centers of the active sites are spaced *d*_cat_ = 0.7 nm from the wall. According to a previous study,^25^ this should maximize the probability that the end of the molecule returns to its origin, increasing the probability of ring closure. The catalyst model has many parameters that could potentially influence the selectivity. For simplicity, we chose a size for the core that is similar to the size of the actual catalyst. The active sites are large enough that the reaction event is sufficiently frequent to gather good statistics. The spacing between the active sites was chosen such that the 4-fold ring that occurs during the reaction is not overstretched. The reaction is calibrated by parametrizing the angular binding potential of the substrate oligomers, as discussed above.

For the number of catalysts in the pore, in order to make the different systems comparable, we chose a constant catalyst surface density for all pore types at σ_cat_ = 0.036 nm^–2^. Due to the curvature of the cylindrical pore, we chose not to measure the surface density at the pore wall but rather at the place where all active sites are located. This corresponds to a distance of *d*_cat_ from the curved wall. This allows for a better comparison between the slit and cylindrical pores.

The flat wall system is depicted in Figure 2b. The volume coupled to the reservoir starts at a distance of *d* = 9 nm perpendicular to the boundary. This system will provide insight into the interfacial effects that occur in this and the two following systems. All wall surfaces are treated as analytical walls, meaning that there are no explicit wall atoms and the interaction between the wall and the molecules is treated by a purely repulsive WCA potential with the length scale σ = 1.535 Å and the energy scale ϵ = 0.833*k*_B_*T*. This approach is also followed in the pore systems.

The slit pore system (Figure 2c) consists of a finite slit pore with a length *l* = 35.8 nm. The width of the pore in the periodic direction is *L*_*y*_ = 10.21 nm. The catalysts are located at a fixed location inside. We varied the distance between the two walls between 2 nm ≤ *d*_slit_ ≤ 6 nm. Due to the constant pore surface area of these different runs, the number of catalysts in the pore was kept constant at *N*_slit_ = 13. For the slit pore and the flat wall system, we distributed the catalysts randomly across the surface, however with the constraint that two catalysts must be separated by at least *l*_ex_ = 2.5 nm.

The cylindrical pore system (Figure 2d) is the one most similar to the experimental setup (by Ziegler et al.). Here, we employ a finite cylindrical pore that is open at both ends. The length of the pore itself is *l* = 35.8 nm. We varied the radius between 1.15 nm ≤ *r* ≤ 3.15 nm. To extrapolate to the limit of large pores, we also ran a simulation with *r* = 6.15 nm. In the small cylindrical pores (*r* = 1.15 nm), we have only very few catalysts inside (*N* = 3). Choosing the distribution of the catalysts at random may position them all close to the entrance of the center. This, in turn, will have an influence on the selectivity. To avoid this, we distributed the catalysts at an equal spacing in the longitudinal axis but still chose the angular coordinate at random. While in the experimental setup of localizing the catalyst inside of the pore, it may also happen that some of the catalysts were tethered to the outside walls of the pore;^39^ however, in our simulations, we handle these exceptions by the investigation of flat wall systems.

The simulation box also consists of a volume outside the pore that spans *L*_*x*_ = 34.2 nm in the longitudinal direction and *L*_*y*_ = *L*_*z*_ = 10.21 nm in the other two directions. Within this volume, GCMC moves can take place; however, we also added padding of *L*_pad_ = 5 nm away from the pore entry to account for diffusional effects.

The substrate density in this reservoir in the simulations is chosen to match the experiments by Ziegler et al. with 0.015 molecules per nm^3^. At this low density, molecules on average are not close enough to interact when they are added or removed. This allowed us to treat the substrate as if it were an ideal gas. However, due to intramolecular interactions, the internal energy of the inserted chains is nonzero. Therefore, the inserted molecules must be in a configuration that follows Boltzmann statistics. We obtained these configurations by sampling the configurations of a single molecule. For each inserted molecule, one of these configurations is randomly selected.

Since the intramolecular energy is accounted for from the collected samples and the intermolecular energy is negligible due to the low density (Δ*U* ≈ 0), we can assume that the chemical excess potential μ_substrate_ = 0. The resulting equilibrium substrate densities are ρ_22_^bulk^ = 0.0145 nm^–3^ and ρ_29_^bulk^ = 0.0142 nm^–3^, which is about 5% less than the concentration in the experiments. This deviation is caused by an underestimation of the chemical potential by assuming that the interaction energy of the substrate oligomers is zero.

One thing worth mentioning is that, due to the different interactions present, the diffusivity of the chains or particles is usually lower inside a confining porous media than in bulk.^40,41^ However, for our simulations, we use a Langevin thermostat with a globally constant friction coefficient, which, together with the low oligomer concentration studied, leads to negligible changes in the observed diffusivity.

Each system examined in this study was simulated using *N* = 8 independent simulation runs, and the average values were used for analysis. The marked errors in the graphs are the standard deviation over all runs. The run time of the simulation was not prescribed, but rather the simulation was interrupted once the error bars were considered small enough. The system temperature was set to *T* = 300 K by using a Langevin thermostat. A more detailed list of the simulation parameters can be found in the Supporting Information.

## 3 Results and Discussion

As the results and conclusions for the two different investigated substrates are very similar, we here discuss only the system with *N*_m_ = 22. All figures showing simulation results for the longer substrate with *N*_m_ = 29 can be found in the Supporting Information.

### 3.1 Homogeneous Reaction

**Figure 4:**
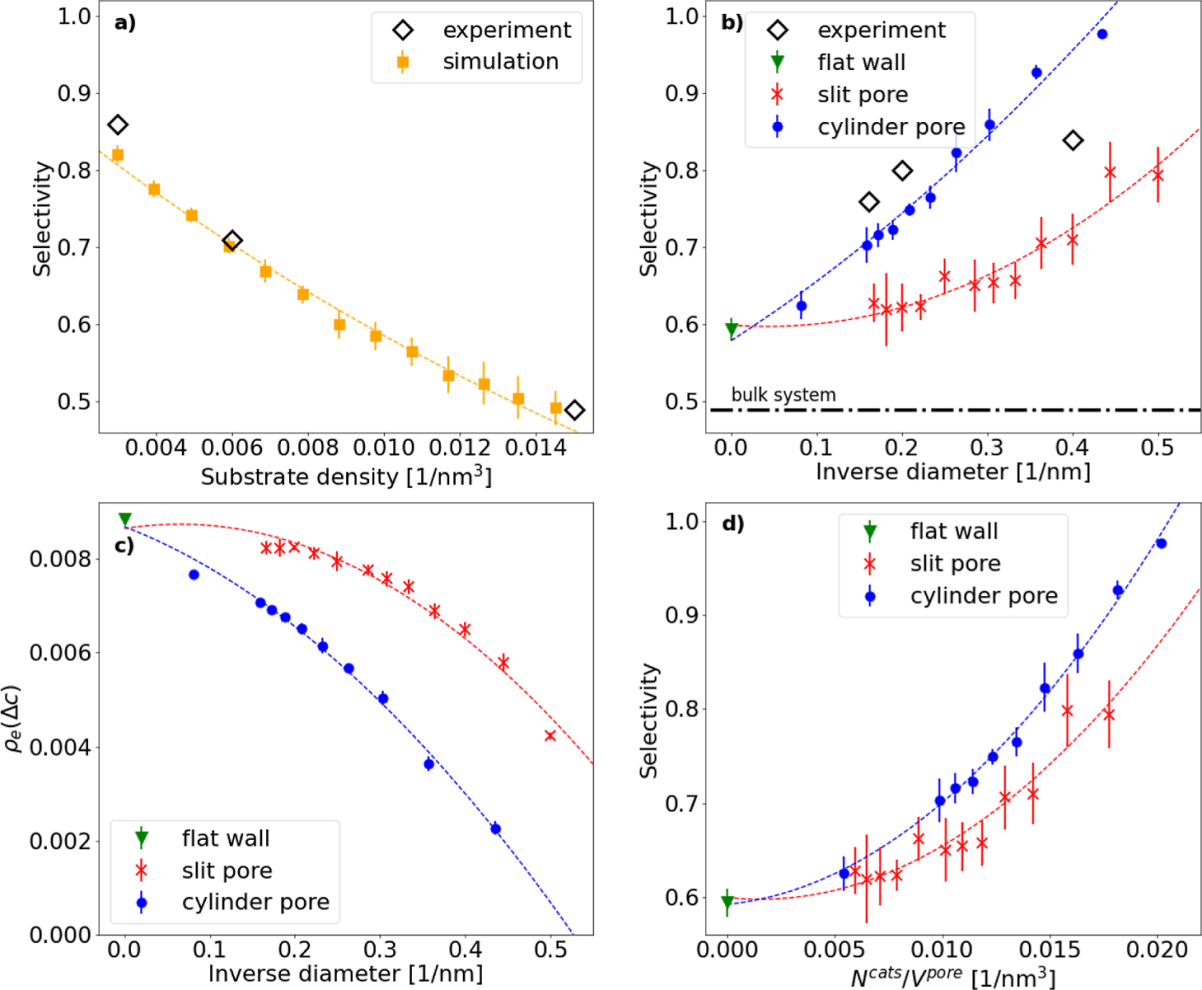
Selectivities and densities obtained from simulations for the substrate of length *N*_m_ = 22. Experimental values are taken from Ziegler et al.^24^ Fitting functions were chosen as a guide to the eye. Error bars shown here are the standard deviations over independent runs. (a) Density dependence of the homogeneous reaction. The fitting curve is an exponential function. (b) Selectivity against the inverse pore diameter/slit width. The result of the bulk reaction at the same reservoir substrate concentration is indicated by the dashed–dotted line. The fitting function for this and the following plots are second-degree polynomials. (c) Local density of the substrate ends at the region of the catalyst ρ_e_(Δ*c*) versus the inverse pore diameter. The region of the catalyst is defined as the position of the active centers with an added margin; see the text for details. (d) Selectivity versus catalyst density inside the pore.

We first investigated the dependence of the substrate concentration on ring-closing selectivity. A decrease in the concentration leads to a lower probability that a molecule from the solution will collide with a catalyst. This will subsequently increase the residence time that a molecule is bound to a catalyst, which in turn increases the time a substrate has for ring closure. The resulting data of the substrate concentration dependence on the ring-closing selectivity is shown in Figure 4a. It clearly demonstrates that lower densities lead to higher ring-closing selectivity. Our results are in excellent agreement with the experimental data by Ziegler et al.^24^ and thus validate the calibration of the simulation model.

### 3.2 Reactions inside Confined Spaces

We now place the catalysts into systems that are bound by a single flat interface, inside slit pores of different widths, and cylindrical pores of different radii in order to investigate varying degrees of confinement: interfacial effects, the presence of confining surfaces, and geometrical curvature effects. In Figure 4b, we report the measured selectivities of our simulations. In detail, we plot the selectivities against the inverse diameter of the pores in order to highlight the convergence toward the limiting case of a flat wall, which is reached in the limit *d* → ∞. The selectivity significantly increases with an increasing degree of confinement. Interestingly, we note that the pure planar interface effect emerging from a flat wall alone increases the ring-closing selectivity already from 49 to 58%. Investigating a slit pore, where we added a second surface at some distance away, further enhances the selectivity depending on the width of the pore. This way, we achieved selectivities ranging from 63% for *d* = 6.0 nm up to 79% for *d* = 2 nm. Moving further from a slit to a cylindrical pore, curvature effects come into play: the selectivity changes from 70% for a diameter of *d* = 6.3 nm up to 97.7% for *d* = 2.3 nm. We additionally simulated a cylindrical pore with *d* = 12.3 nm in order to bridge the gap in the case of a flat wall. If we plot the simulated selectivity against the inverse diameter, then the selectivity increase is found to be almost linear for the cylindrical pores (Figure 4b). Importantly, both the cylindrical and the slit pore selectivities converge toward the flat planar interface, which results in the case of large diameters.

While the simulations slightly underestimated the experimental values for the larger pore sizes (*d* ∈ {6.3 nm, 5.0 nm}), the selectivity was overestimated for smaller pores. The main reason for this is that our model does not include any specific polymer–wall interactions that occur between the ester compounds and the silica pore. Since the goal of this study is not to investigate chemistry-specific effects, we explicitly decided not to include this kind of interaction in our approach, which might explain some of the deviations between the simulated and experimental values. More in detail, in our model we have only steric and therefore repulsive interactions included; thus, there are only a few factors that can cause confinement effects. To shed further light on this, we investigated the local density profile of the end-monomers, ρ_e_(Δ*c*), of the substrate as a function of the distance to the wall. The end-monomer density instead of, e.g., the center of mass density was chosen since only the ends can bind to the catalyst. The distance range away from the surface, which we measured was the position of the reactive centers plus/minus the catalyst size, correspondingly, Δ*c* = (0.7 ± 0.3) nm. In Figure 4c, this local density is plotted against the inverse diameter. Classical polymer theory predicts that the oligomer density decreases near an impenetrable wall for entropic reasons. This can be observed in our simulations in the case of a flat wall, where the density near the wall, ρ_e_^flat^ = 0.0089 nm^–3^, is much lower than far from the wall, ρ^bulk^ = 0.0145 nm^–3^. This effect is even more pronounced inside a pore, where two interfaces or a curved boundary act on the substrate. For a decreasing pore size, the reduction in the local density around the catalyst is increased. Additionally, due to curvature effects, the cylindrical pores have a lower local density than the slit pores with the same diameter.

The local end-monomer density for the slit pores (red data in Figure 4c) appears to saturate for small inverse pore widths, yet this saturating local density in the large pores is lower than the local density near a single flat wall (green triangle in Figure 4c). This can be explained by the fact that the system is not in diffusive equilibrium, i.e., there is a substrate density gradient along the longitudinal pore axis due to the drainage of the substrate during the reaction. We tested this hypothesis by measuring the local density of a slit pore system in which there were no added catalysts and thus no reactions taking place. In that case, the local density for large slit pores recovered the same value as in the flat wall case. Although the observed density gradient along the pore axis in our simulations is partially due to the increased reaction rate in the simulations compared to the experimental olefin metathesis, such effects always appear in the case of fast reactions or in sufficiently long pores, which depending on the material can easily extend to the micrometer scale.

In general, comparing cylindrical and slit pores at the same diameter can be misleading since volume and surface area scale differently for the two types of pores. For our simulations, where we varied the pore size, we used a constant catalyst surface density to eliminate the different surface scales. In order to elucidate the effect of the catalyst volume density  on the selectivity, we present the corresponding simulation data in Figure 4d. This representation allows for the comparison of confinement and surface effects at the same volume and surface density of the catalyst. Our simulation results reveal—as expected—a strong increase of the selectivity with increasing catalyst volume density; however, the cylindrical pores at the same values  reveal selectivities that are about 10% higher for the cylindrical pores than for the slit pores. Since the simulations are performed at a fixed catalyst surface density, relating the pore volume to a degree of confinement allows identification of this difference in selectivity with curvature effects.

**Figure 5:**
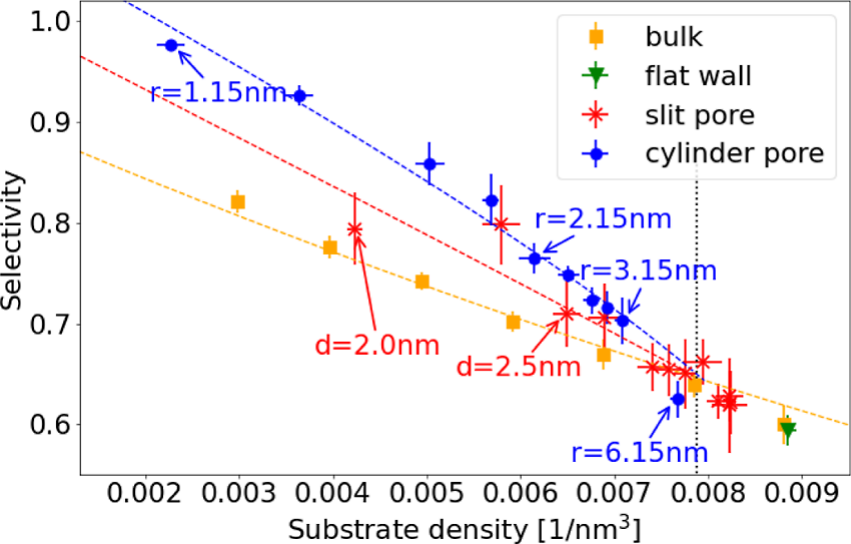
Selectivity vs local density of the substrate ends. Smaller pores show lower local substrate densities. Bulk here refers to the homogeneous reaction at the given substrate density. For small pores, the measured selectivity is larger than the selectivity of a homogeneous reaction at the same density and cylindrical pores reveal a higher selectivity compared to slit pores. The vertical dotted line marks the upper bound of the region, where confinement effects are observed, i.e., data for bulk, in the pores and at a planar interface become indistinguishable.

Finally, our simulations allow for correlating the obtained local end-monomer density and selectivity for the different systems examined. In the limit of large pores (right data in Figure 5), the selectivity increase in pores perfectly coincides with the change of the substrate density in bulk (yellow data) and also with the selectivity increase at a planar interface (green triangle). The vertical dashed line in Figure 5 indicates the limit where a direct proportionality to the density change is observed, i.e., the transition to confinement effects. For the parameters investigated here, this transition occurs roughly at *r* ≈ 6 nm for cylindrical pores and *d* ≈ 3 nm for the slit pores. For smaller pores, the measured selectivity outperforms the bulk reaction at the same density, implying that the local end-monomer density cannot be the only source for the selectivity increase. Confinement must therefore directly affect the ring closure process by reducing the number of possible configurations for the substrate. Again, the increase is more pronounced for cylindrical pores than for slit pores, revealing that curvature amplifies this effect, i.e., the configurational degrees of freedom of the substrate at a curved interface are reduced much stronger than at a planar interface, thus enhancing the ring-closing probability. As an additional measurement, we determined the mean end-to-end distance (see Figure S6 in the Supporting Information). As could be expected, the end-to-end distance within the small cylindrical pores decreased with decreasing pore size. Since the geometrical restriction is only acting in one direction for the slit pores, the reduction in the end-to-end distribution is less pronounced for these pores compared to the cylindrical pores with the same diameter.

## 4 Conclusions

In this paper, we have developed a coarse-grained model for studying a generic catalytically activated ring-closing polymerization reaction near an unstructured catalyst. Using particle-based grand-canonical MD simulations and a collision-based reaction mechanism, we parametrized our model to reproduce the bulk ring-closing selectivities of a diene metathesis reaction studied in a recent work by Ziegler et al.^24^ This was done for substrate polymers consisting of *N*_m_ = 22 and *N*_m_ = 29 monomers. For the homogeneous reaction, we recovered the expected reduction of the ring-closing selectivity with increasing substrate density. While this is a well-known fact, it demonstrates the validity of our modeling approach. Using this model, we performed an extensive investigation of the effects of the presence of a wall near the catalysts. In accordance with our previous analytical work,^25^ we also found with our simulational approach that the presence of a wall enhances the excess return probability of chain ends, which leads to a corresponding change in the ring-closing probability due to the wall constraint. For all systems investigated in the present work, we could measure an increase in the ring-closing selectivity compared to the selectivity measured in a homogeneous system at the same substrate reservoir density. We quantified this wall effect and demonstrated that it stems from the reduction of the density of substrate chain ends near the catalysts.

We then continued to investigate slit pores and cylindrical nanopores of various sizes to systematically disentangle confinement effects from wall-induced effects. Interestingly enough, there is a further increase in ring-closing selectivity if we geometrically confine the catalysis. The only difference we noted between the two investigated oligomer lengths was that noticeable confinement effects occurred for the larger oligomer at larger pore sizes when compared to the shorter oligomer lengths. The geometric-induced selectivity increase stems in part from the fact that the density of chain ends near the catalysts decreases further due to a reduction in overall substrate density within the pore and is a pure confinement effect. Noteworthy, we observed that for small pores, the increase cannot be related to the local density alone but is rather due to two additional effects: (i) if the pore size becomes comparable to the typical length of the substrate, the conformational degrees of freedom are restricted and, thus, the return probability is further enhanced (this can be observed, e.g., for slit pores <3 nm). (ii) The observed selectivity enhancement is much stronger for the cylindrical pores studied, indicating that curvature further enhances the return probability and thus the selectivity. The latter effect increases with the pore curvature, i.e., as 1/*r*, in perfect agreement with the observations from our simulations. The experimental data of the metathesis reaction Ziegler et al.^24^ show good agreement with our observed trends.

Summing up, we can always relate the increase of the ring-closing selectivity to the reduction of substrate density close to the catalyst, which, however, varied for the different systems. In particular, the strong dependence on wall curvature effects was unexpected and could likely be exploited with specially corrugated surfaces that have an optimal shape for the employed substrate particles. With our catalytic coarse-grained model in hand, one could also investigate confinement effects for other pore geometries and substrates and see which kind of geometry would be optimal for the catalytic process.

## Data Availability

The data that support the findings and the scripts to reproduce this study are openly available in DaRUS—the Data Repository of the University of Stuttgart, https://doi.org/10.18419/darus-3642.

## Supporting Information Available

Additional simulation parameters results of the longer substrate with *N*_m_ = 29 (PDF)
