# Supplementary material for: Disentanglement of Surface and Confinement Effects for Diene Metathesis in Mesoporous Confinement
Source: ACS Omega. 2023 Dec 19;9(1):598–606. doi: 10.1021/acsomega.3c06195 (PMC10785312; doi:10.1021/acsomega.3c06195)
Supplement: Supplementary file 1 — ao3c06195_si_001.pdf [file ao3c06195_si_001.pdf]

Supplementary Material: Disentanglement of Surface and Confinement Effects for Diene Metathesis in Mesoporous Confinement

Ingo Tischler,[†] Alexander Schlaich,[‡,†] and Christian Holm[*,†]

†*Institute of Computational Physics, University of Stuttgart, Germany*

‡*Stuttgart Center for Simulation Science, University of Stuttgart, Germany*

E-mail: holm@icp.uni-stuttgart.de

Figures and tables that were left out of the main paper due to some redundancy are found here. Table S1 lists the parameters of our simulations. Figure S1 shows the dependency on the bond angle potential stiffness, which was calibrated to match the experimental values. Figure S2 shows the measured selectivities of the of the different systems in real space. Figure S3 shows the results for the longer substrate $N_m = 29$. Figure S4 plots the selectivity of the system against their measured density. For a better comparison between the two different oligomer lengths we plotted the results together in one plot shown in Figure S5. The last plot (Figure S6) shows the average end-to-end distances for different porous systems at varying pore widths.

Table S1: Parameters used for the simulations.

Parameter	Unit	Value
Time step	Δt	1.18 fs
Temperature	T	300 K
Langevin friction coefficient	γ	39.3 fs^{-1}
LJ energy	ϵ	$0.833k_\text{B}T$
LJ length	σ	0.1535 nm
FENE bond strength	K^FENE	$1061k_\text{B}T$
FENE max distance	r_max	0.2303 nm
Harmonic bond strength	K^Harm	$140k_\text{B}T$
Harmonic bond distance	r_0	0.1339 nm
Bond breakage rate	τ_break^{-1}	0.844 ps^{-1}

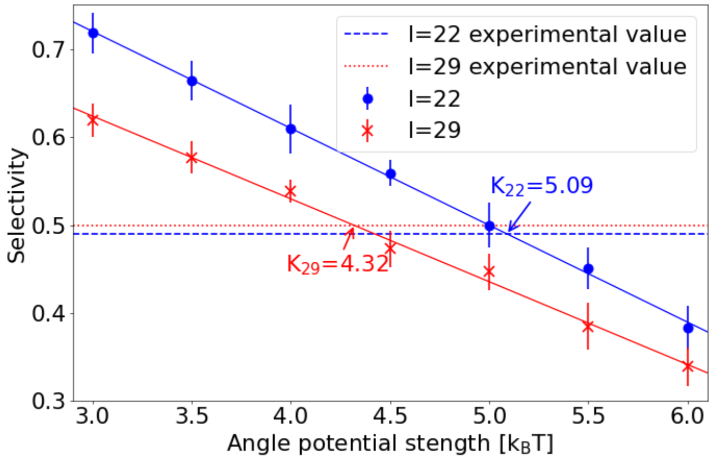

Figure S1: Calibration simulation of the angle bond stiffness, which has been determined to be $K_{22} = 5.09\ k_\mathrm{B}T$ and $K_{29} = 4.32\ k_\mathrm{B}T$.

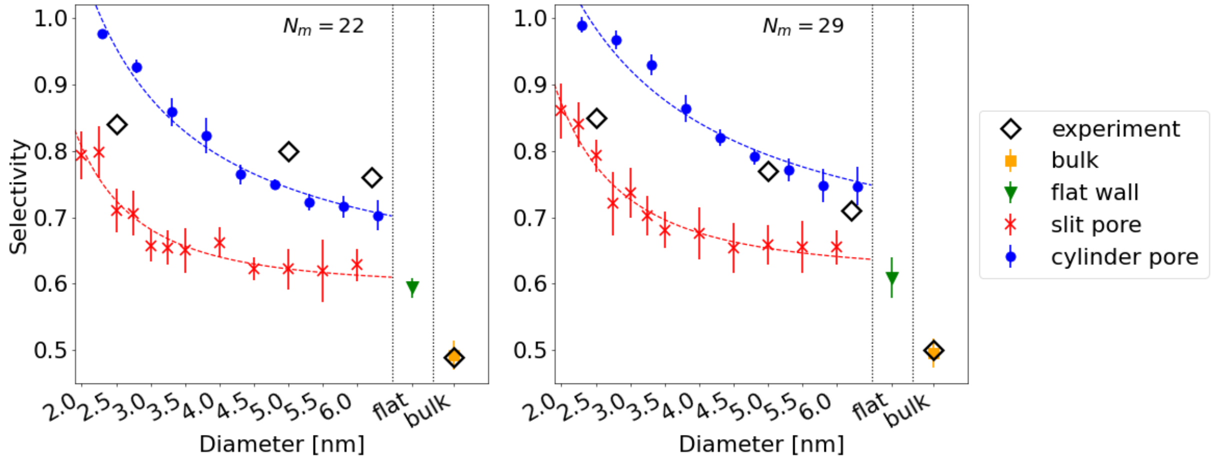

Figure S2: Selectivity vs pore diameter of the different systems. The left plot shows the shorter oligomer. Experimental values taken from.[1] Comparing the results of the two different oligomers reveals a size scaling effect. The larger oligomer shows a slightly larger increase in response from the homogeneous to the flat wall response, as the near surface density depletion zone is larger for longer oligomers. It can also be seen that the selectivity increase in the slit pore starts at larger pore diameters.

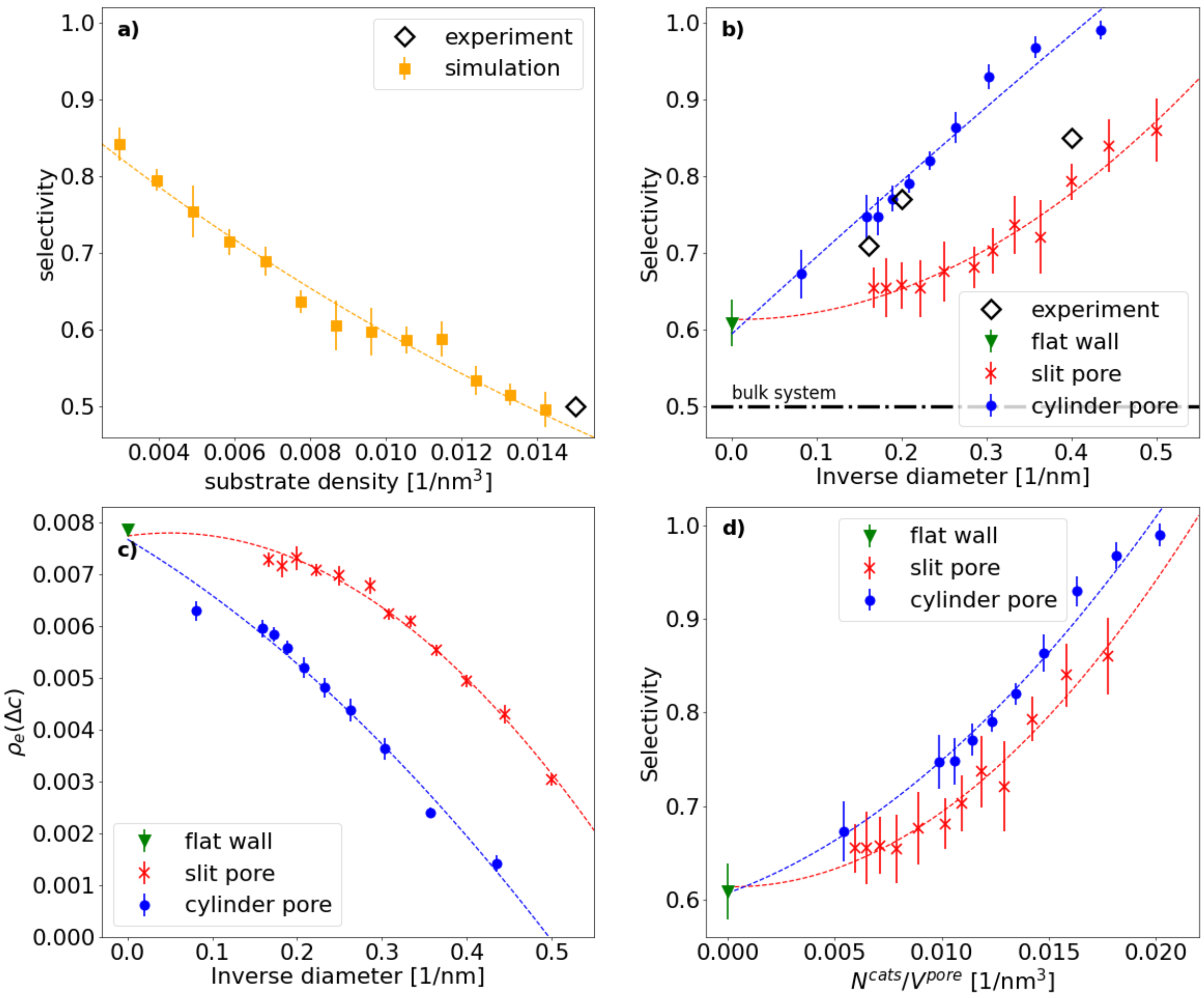

Figure S3: Selectivities and densities obtained from simulations for the substrate of length $N_m = 29$. Experimental values are taken from Ziegler et al.[1]. The fitting functions were chosen as a guide to the eye. The error bars shown here are the standard deviation over independent runs. a) Density dependence of the homogeneous reaction. The fitting curve is an exponential function. b) Selectivity against the inverse pore diameter/slit width. The result of the bulk reaction at the same reservoir substrate concentration is shown in the dotted (simulation) and dash dotted (experiment) line. The fitting function for this and the following plots are second degree polynomials. c) Local density of the substrate ends at the region of the catalyst $\rho_e(\Delta c)$ vs the inverse pore diameter. The region of the catalyst is defined as the position of the active centres with an added margin. So measured from the wall the region is: $\Delta c = (0.7 \pm 0.3)$ nm. d) Selectivity vs the catalyst density inside the pore.

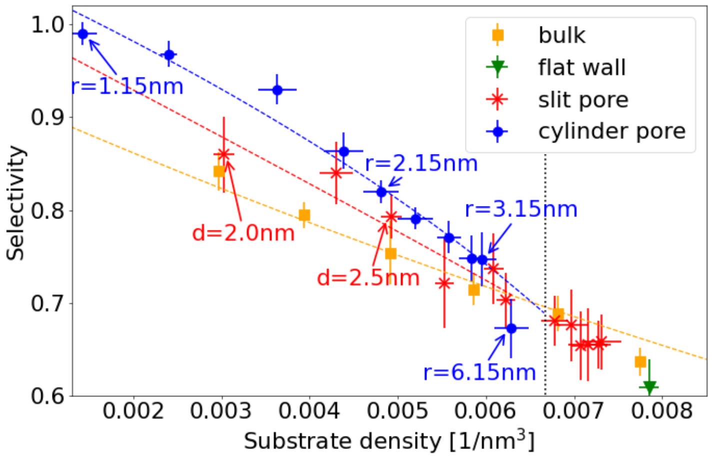

Figure S4: Selectivity vs. local density of the substrate ends ($N_m = 29$). Smaller pores show lower local substrate densities. Bulk here refers to the homogeneous reaction at the given substrate density. For small pores the measured selectivity is larger than the selectivity of a homogeneous reaction at the same density and cylindrical pores reveal a higher selectivity compared to slit pores. The vertical dotted line marks the upper bound of the region, where confinement effects are observed, i.e. data for bulk, in the pores and at a planar interface become indistinguishable.

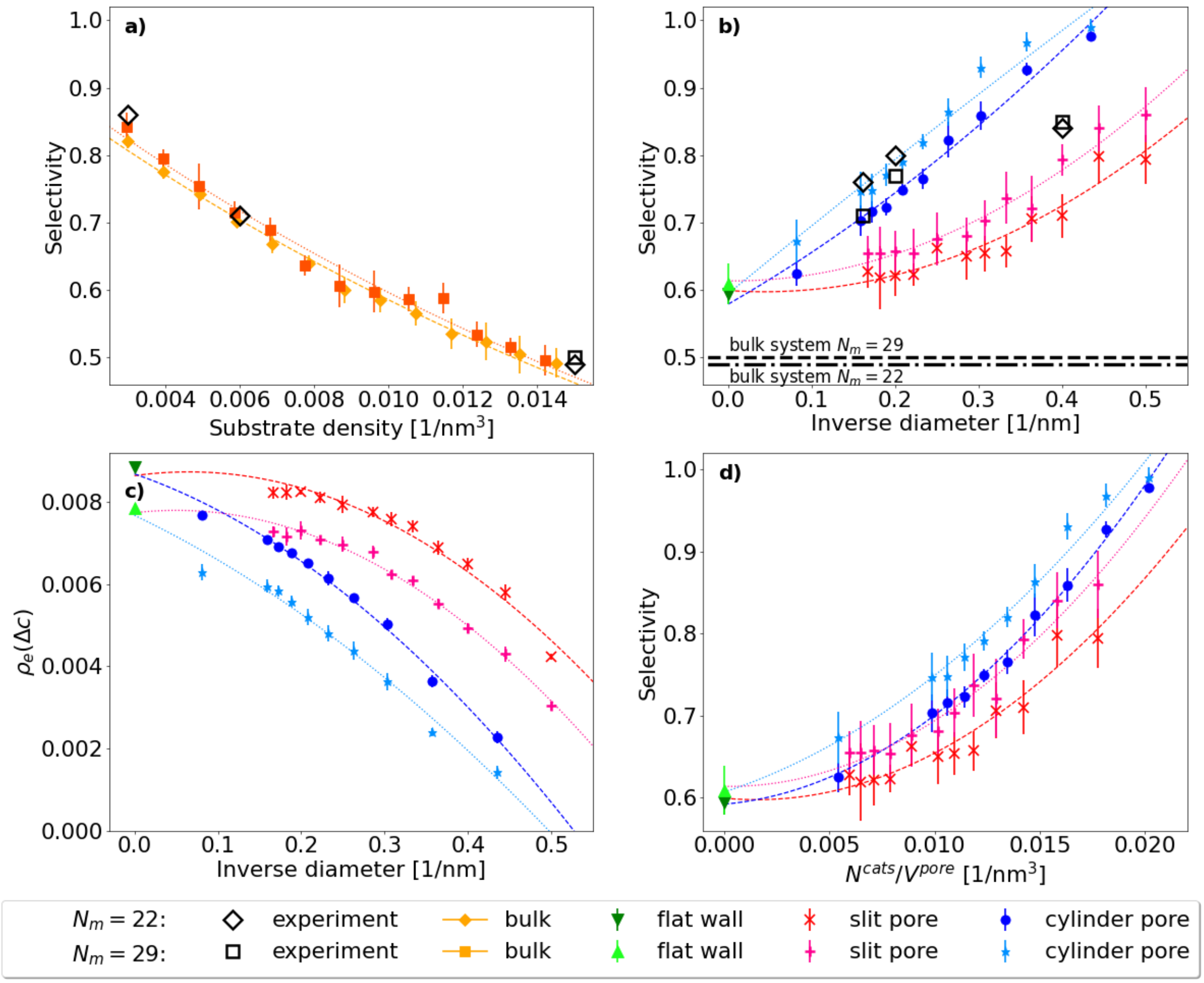

Figure S5: Plots combining the results of the simulations of both oligomer lengths. This shows that confining effects affect the longer oligomer slightly more. Experimental values are taken from Ziegler et al.[1]. a) shows the selectivity scaling of the homogeneous reaction with the substrate density. b) plots the selectivity against the inverse pore diameter for cylindrical and slit pores. c) Local density of the substrate ends at the region of the catalyst $\rho_e(\Delta c)$ vs the inverse pore diameter. And d) plots the selectivity vs the catalyst density inside the pore.

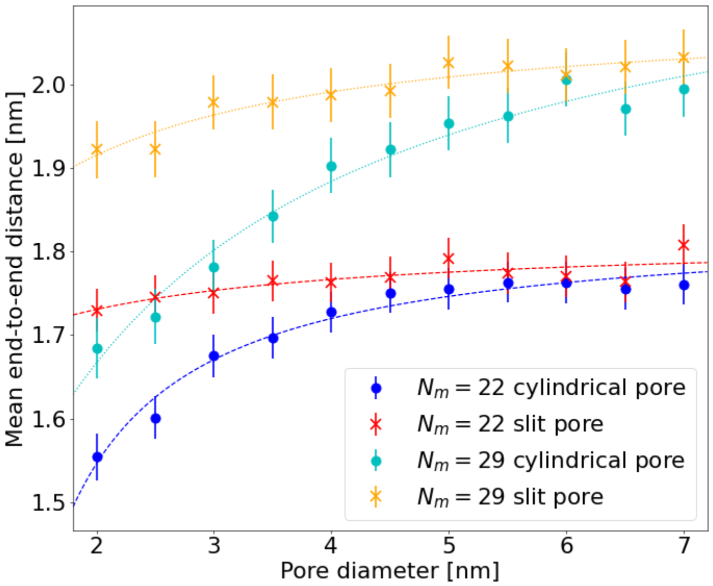

Figure S6: Average end-to-end distances of the oligomers within pores of different diameters. These measurements were done without any catalysts located inside of the pores. The chosen fit function is: $f(x) = a/(x + b) + c$

References

(1) Ziegler, F.; Kraus, H.; Benedikter, M. J.; Wang, D.; Bruckner, J. R.; Nowakowski, M.; Weißer, K.; Solodenko, H.; Schmitz, G.; Bauer, M. et al. Confinement Effects for Efficient Macrocyclization Reactions with Supported Cationic Molybdenum Imido Alkylidene N-Heterocyclic Carbene Complexes. *ACS Catalysis* **2021**, *11*, 11570–11578.
